# Effectiveness of freezing temperatures on dormancy release of temperate woody species

**DOI:** 10.1093/aob/mcae112

**Published:** 2024-07-25

**Authors:** Huanjiong Wang, Wenrui Bai, Zhi Hu, Shaozhi Lin, Quansheng Ge

**Affiliations:** Key Laboratory of Land Surface Pattern and Simulation, Institute of Geographic Sciences and Natural Resources Research, Chinese Academy of Sciences, Beijing 100101, China; Key Laboratory of Land Surface Pattern and Simulation, Institute of Geographic Sciences and Natural Resources Research, Chinese Academy of Sciences, Beijing 100101, China; University of Chinese Academy of Sciences, Beijing 100049, China; Key Laboratory of Land Surface Pattern and Simulation, Institute of Geographic Sciences and Natural Resources Research, Chinese Academy of Sciences, Beijing 100101, China; University of Chinese Academy of Sciences, Beijing 100049, China; Key Laboratory of Land Surface Pattern and Simulation, Institute of Geographic Sciences and Natural Resources Research, Chinese Academy of Sciences, Beijing 100101, China; University of Chinese Academy of Sciences, Beijing 100049, China; Key Laboratory of Land Surface Pattern and Simulation, Institute of Geographic Sciences and Natural Resources Research, Chinese Academy of Sciences, Beijing 100101, China

**Keywords:** Chilling, forcing, climate change, phenology, budburst, dormancy, temperate woody species

## Abstract

**Background and Aims:**

Spring phenological change of plants in response to global warming may affect many ecological processes and functions. Chilling temperature regulates budburst date by releasing dormancy. However, whether freezing temperatures (<0 °C) contribute to dormancy release remains of debate. Our poor understanding of the role of chilling makes estimating shifts in budburst date difficult.

**Methods:**

A 2-year chilling–forcing experiment was explicitly designed to test the effects of chilling temperatures on dormancy release of nine temperate woody species in Beijing, China. A total of 1620 twigs were first exposed to a wide range of temperatures (−10 to 10 °C) with different durations and then moved to growth chambers. Based on budburst data in experimental conditions, we examined whether freezing temperatures are effective on dormancy release. We also developed a new framework for constructing chilling functions based on the curve between chilling duration and forcing requirement (FR) of budburst. The chilling function derived from this framework was not affected by experimental forcing conditions.

**Key Results:**

We demonstrated that freezing temperatures down to −10 °C were effective in dormancy release. The rate of dormancy release, indicated by the rate of decay in the chilling duration–FR curve, did not differ significantly between chilling temperatures in most cases, although it exhibited a maximum value at 0 or 5 °C. The chilling function-associated phenological models could simulate budburst date from independent experimental and observational data with a mean RMSE of 7.07 d.

**Conclusions:**

The effective freezing temperatures found here are contrary to the well-known assumption of <0 °C temperature generally not contributing to accumulated chilling in many previous chilling functions. A chilling function assuming that temperature below an upper temperature threshold has the same effects on dormancy release could be adopted to calculate chilling accumulation when using experiments to develop spring phenological models based on the chilling–forcing relationship.

## INTRODUCTION

Spring phenology (e.g. budburst, leaf-out and flowering) of plants is an adaptive trait (Wolkovich and Ettinger, 2014). In temperate and boreal regions, budburst date should first minimize the frost risk of plants and then maximize the growing season duration to sustain their productivity (Bennie *et al.*, 2010; Körner *et al.*, 2016; Zohner *et al.*, 2020). Similarly, appropriate flowering timing could help plants match the activity of pollinators and avoid frost damage, ultimately enhancing the probability of reproductive success (Burkle *et al.*, 2013). In the past several decades, shifting towards earlier spring phenology has been reported in many regions of the world, especially in the temperate and boreal regions (Ge *et al.*, 2015; Menzel *et al.*, 2020; Pareja-Bonilla *et al.*, 2023; Pearse *et al.*, 2023). Such phenological changes could affect many ecological processes and functions, such as carbon, water, heat flux between the land surface and atmosphere (Keenan *et al.*, 2014; Hiestand and Carleton, 2019), the relationship between trophic levels (Thackeray *et al.*, 2016), and the distribution of species (Chuine, 2010).

For boreal and temperate species, budburst date in spring is closely related to climatic conditions during preceding periods of dormancy and ontogenetic development. Induced by low temperatures and short daylength in autumn, the apical meristem of plants ceases growth, and terminal buds are observed (Wang *et al.*, 2022*a*). Buds first enter a state of endodormancy, during which physiological conditions prevent growth, and sufficient chilling temperature (low temperatures in the dormant season) is required to break endodormancy (Lang *et al.*, 1987; Delpierre *et al.*, 2016). Subsequently, buds enter a state of ecodormancy during which growth is mainly limited by low temperatures (Lang *et al.*, 1987). With forcing requirements fulfilled by higher temperatures and longer daylength in spring, ontogenetic development begins and finally leads to budburst. However, there is no strict distinction between endo- and ecodormancy because the release of endodormancy is progressive, and the effects of chilling and forcing overlap during a certain period in early spring (Harrington and Gould, 2015; Wang *et al.*, 2023). Hereafter, we use the term dormancy to represent a general state of bud rest (including both endodormancy and ecodormancy).

The effects of climate warming on spring phenology of boreal and temperate trees could be predicted by process-based phenological models. These models quantified the process of dormancy release by a chilling function and ontogenetic development by a forcing function (Chuine *et al.*, 2016). Chilling accumulation (calculated by chilling functions) in winter could decrease the forcing requirements (calculated by forcing functions) of budburst (Murray *et al.*, 1989; Chuine *et al.*, 1999). The accumulated forcing calculated from different forcing functions correlated positively and significantly with each other (Wang *et al.*, 2020*a*). Thus, using different forcing functions almost did not affect phenological prediction. However, chilling functions assume that the rate of dormancy release is a function of chilling temperature (Chuine, 2000). Recently, many phenological studies have used chilling functions to quantify the chilling requirements of trees or forecast spring phenology under future climate change scenarios (Ettinger *et al.*, 2020; Wang *et al.*, 2022*b*), but the choices of chilling functions would affect the results. For example, when using the Utah function (ADN in Table 1) to measure chilling, warming may increase accumulated chilling (e.g. temperature <1.4 °C increases to >1.4 °C), but warming always reduces accumulated chilling when using the function assuming freezing temperatures are effective (ASF in Table 1).

**Table 1.** Summary of chilling functions developed by previous studies. *T*_lower_ and *T*_upper_ represent the lower and upper threshold for effective chilling, respectively.

Type	Code	*T*_lower_ (°C)	*T*_upper_ (°C)	Reference
Functions based on assumed temperature response	ADN	1.4	15.9	(Richardson *et al.*, 1974; Luedeling *et al.*, 2009)
	ASN_1_	0	7.2	(Weinberger, 1950; Luedeling *et al.*, 2009; Rezazadeh and Stafne, 2018)
	ASN_2_	0	5	(Fu *et al.*, 2015; Ma *et al.*, 2018)
	ASF	−∞	5	(Cannell and Smith, 1983; Du *et al.*, 2019; Wang *et al.*, 2022*b*)
	ADF	−4.7	16	(Harrington *et al.*, 2010)
Functions based on inverse modelling	IDF_1_	−23	18	(Caffarra *et al.*, 2011)
	IDF_2_	−2	12	(Lin *et al.*, 2022)
	IDN	3	17	(Chuine, 2000)
Functions based on experimental data	EDF_1_	−3.4	10.4	(Sarvas, 1974; Harrington *et al.*, 2010)
	EDF_2_	−3.4	20	(Zhang *et al.*, 2022)

First letter of the model code: A, assumed response; I, inverse modelling; E, experiments. Second letter of the model code: D, different response within effective temperature range; S, same response within effective temperature range. Third letter of the model code: F, freezing temperature (< 0 °C) is effective; N, freezing temperature is noneffective.

Previous studies have used three ways to determine chilling functions. Several studies used an assumed temperature response based on fragmentary experimental evidence and climate background of the study area. For example, the Utah model assumes that temperatures between 1.4 and 15.9 °C affect dormancy release differently (Richardson *et al.*, 1974). The second widely used method is inverse modelling, which assumes a fixed form of chilling function and fits the function parameters statistically using long-term observational data. The temperature response from assumption and inverse modelling methods often generates biologically unrealistic models (Hänninen *et al.*, 2019). The last and the most effective method for determining chilling functions is to use controlled experiments specifically designed to examine the temperature response of dormancy release. For instance, Sarvas (1974) revealed the temperature response of dormancy release for *Betula pubescens* seedlings and found that chilling temperature was most effective at 3.5 °C. More recently, Zhang *et al*. (2022) experimentally determined the chilling function for four subtropical trees. Two other studies used controlled experiments to study the changes in forcing requirement or number of days to budburst under different chilling conditions but did not focus on constructing chilling functions (Myking and Heide, 1995; Baumgarten *et al.*, 2021).

**Fig. 1.**
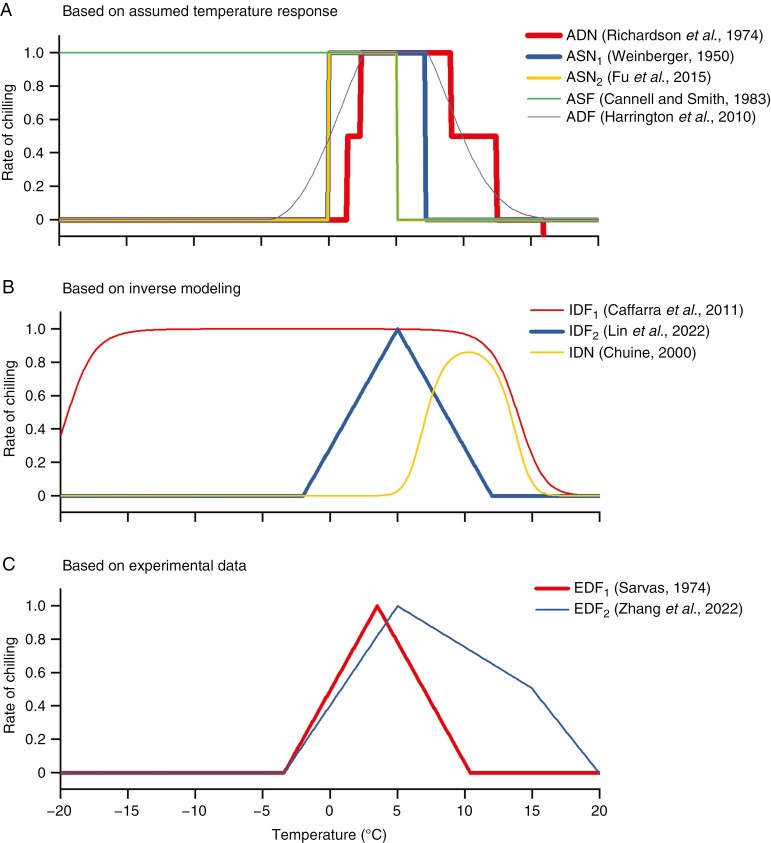
Curves of chilling functions proposed by previous studies. The reference and the upper/lower threshold of each chilling function are shown in Table 1.

Chilling functions derived from three types of methods differ significantly in model structures (Fig. 1) and lower/upper temperature thresholds (Table 1). We coded existing chilling functions based on their model determination methods (A, assumed response; I, inverse modelling; E, experiments), effects of different temperatures (D, different response within effective temperature range; S, same response within effective temperature range) and lower temperature thresholds (F, freezing temperature is effective; N, freezing temperature is not effective). Several studies assumed the same effect within the effective temperature range, including ASF (Cannell and Smith, 1983), ASN_1_ (Weinberger, 1950) and ASN_2_ (Fu *et al.*, 2015), but others assumed that an optimal temperature or temperature range exists, such as ADF (Harrington *et al.*, 2010), ADN (Luedeling *et al.*, 2009) and IDN (Chuine, 2000). Temperatures slightly above zero and below an upper temperature threshold are always included in the effective range (Table 1). However, in four of ten chilling functions (Table 1), the freezing temperature does not contribute to accumulated chilling (lower temperature threshold <0 °C).

Because understanding of the physiology and process of chilling effects on the rate of dormancy release was limited and most of the existing chilling functions were not determined based on the empirical evidence, any current observations of changes in chilling accumulation – and all forecasts with warming – are uncertain (Wolkovich *et al.*, 2022). Thus, controlled experiments investigating the effective temperature range in dormancy release are urgently needed. Recently, Zhang *et al*. (2021) and Baumgarten *et al*. (2021) found that −2 °C was effective on dormancy release. However, whether a lower temperature (−10 to −5 °C), which frequently occurs in winter of temperate regions, is still effective in dormancy release needs to be further tested. In this study, we carried out an experiment on dormancy release of nine common temperate species in northern China. We exposed the twigs of these species to artificial chilling temperatures (−10 to 10 °C) with different lengths to address the following questions: (1) whether freezing temperatures far lower than 0 °C could effectively release buds from dormancy and (2) whether different chilling temperatures have similar or significantly distinct effects on dormancy release.

## MATERIALS AND METHODS

### Study species and site

We selected nine deciduous species commonly distributed in temperate regions of China (Supplementary Data Table S1), including three trees and six shrubs. The twigs of these species were sampled at the Olympic Forest Park in Beijing (40°01ʹ3.00″N, 116°23ʹ2.98″E). The sampling site belongs to a typical temperate monsoon climate, with warm and humid summer (mean temperature of 26.48 °C and accumulated precipitation of 332.18 mm during June–August), and cold and dry winter (mean temperature of −0.82 °C and accumulated precipitation of 11.16 mm during December–February). In winter, the daily minimum temperature is frequently lower than −5 °C or even lower than −10 °C around 1 January (Supplementary Data Fig. S1).

The daily mean, maximum and minimum temperature data in Beijing (1951–2022) were derived from the China Meteorological Data Service Center (http://data.cma.cn/en). Because hourly temperature data were unavailable, we generated hourly temperatures based on a sine function (Zohner *et al.*, 2020).

### Twig experiments

For each species, we selected five healthy and mature individuals for collection of twigs (i.e. five replicates for each species). On 12 November 2020, 16 twigs (containing three to six winter buds) with a length of 20–30 cm were collected from each individual. For each species, the collected 80 twigs were randomly divided into 16 groups (each group containing five twigs from different individuals). Among them, 15 groups were moved to five refrigerators in full darkness (three groups in each refrigerator) with different temperature settings (−15, −10, −5, 0 and 5 °C). Before moving them to refrigerators, we sealed the cut ends of twigs with paraffin to prevent water loss and stored them in plastic bags. The remaining group was placed in a 0.25-L glass bottle with tap water and moved to growth chambers (produced by Ningbo Jiangnan Instrument Factory). The temperature of the growth chambers was set at 25 °C under light (100 μmol m^−2^ s^−1^) lasting for 14 h, and at 15 °C in the dark lasting for 10 h periodically. For the samples stored in refrigerators, we took out one group of twigs after 13, 30 and 60 d, and placed them in growth chambers with the same temperature and photoperiod settings. On 12 December 2020 and 11 January 2021, a group of twigs were collected in the field and moved to growth chambers to monitor phenology. On 12 December 2020, we additionally collected five groups of twigs for each species and placed them in the five refrigerators mentioned above. These samples were moved to growth chambers after 30 d (on 11 January 2021). The additional samples were used to compare the effects of natural chilling (in the field) and artificial chilling (in the refrigerators).

Because the sample size (three chilling treatments) from the 1-year experiment was not enough to develop chilling functions, we conducted a similar experiment in the next year. In the first-year experiment, the twigs were almost dead when moving from −15 °C to forcing conditions (possibly due to lack of cold acclimation). Therefore, in the second-year experiment, we removed the chilling treatment of −15 °C but added a chilling treatment of 10 °C. Meanwhile, we collected twigs 10 d later than the first year, because 10 d later could increase the cold hardiness of twigs by −3 °C on average based on data from five species in Beijing (Hu *et al.*, 2022). On 22 November 2021, 105 twigs with a length of about 20–30 cm were collected for each species (from the same individuals in 2020), and the collected samples were randomly divided into 21 groups (each containing five twigs). Among them, 20 groups were moved to five refrigerators (four groups in each refrigerator) with different chilling temperatures (−10, −5, 0, 5 and 10 °C), while the remaining group was placed in a 0.25-L glass bottle with tap water and moved to growth chambers where the temperature and photoperiod were set the same as the previous year. After 2, 4, 8 and 12 weeks, a group of twigs was taken from refrigerators and moved to growth chambers to monitor phenology. On the same dates, five twigs for each species were collected in the field and moved to growth chambers. A summary of the 2-year experiment is shown in Table 2 and Supplementary Data Fig. S1.

**Table 2.** Summary of the two-year chilling experiment in this study

First-year experiment		Second-year experiment		Usage
Sampling dates	Chilling treatment	Sampling dates	Chilling treatment	
12 Nov. 2020	No extra chilling 13 d extra chilling (5 × *T*)* 30 d extra chilling (5 × *T*) 60 d extra chilling (5 × *T*)	22 Nov. 2021	No extra chilling 14 d extra chilling (5 × *T*) 28 d extra chilling (5 × *T*) 56 d extra chilling (5 × *T*) 84 d extra chilling (5 × *T*)	Training
12 Dec. 2020	No extra chilling	6 Dec. 2021	No extra chilling	Testing
	30 d extra chilling (5 × *T*)†	20 Dec. 2021	No extra chilling	
11 Jan. 2021	No extra chilling	17 Jan. 2022	No extra chilling	
		14 Feb. 2022	No extra chilling	

5 × T represents that the twigs were chilled at five temperature conditions. In the first-year experiment, the five temperatures were −15, −10, −5, 0 and 5 °C, while in the second-year experiment, the five temperatures were −10, −5, 0, 5 and 10 °C. Training and testing: data used to train or test the chilling functions.

*At this date, in order to test the potential frost damage, only the twigs at −15 and −10 °C were moved to forcing conditions.

†These samples were used to compare the effects of natural chilling (in field) and artificial chilling (in refrigerators) on dormancy release.

When twigs were moved into growth chambers, we immediately recorded the total number of buds in each twig and monitored the number of buds in a specific phenological event once per 3 d over the next 90 d. Each twig was recut weekly to avoid xylem embolism. We observed three phenological events based on criteria of the BBCH (acronyms in Germany: Biologische bundesanstalt, Bundessortenamt und CHemische industrie) scale (Meier, 2001; Taghavi *et al.*, 2022). BBCH 9 is defined as the buds showing green tips. BBCH 11 is defined as the buds developing an unfolded leaf. BBCH 60 is defined as the buds developing an opened flower. For each twig, the proportion of budburst was defined as the number of buds that had reached BBCH 9 after 90 d (since the date they were moved to forcing conditions) divided by the total number of buds (the proportion of budburst was only monitored in the second-year experiment). We also recorded the number of twigs that could not reach the BBCH 9 after 90 d and calculated the proportion of survived twigs in each treatment (Supplementary Data Table S2). Three species first entered the stage of BBCH 60 and then BBCH 11, while the other species unfolded leaves earlier than flowering (Supplementary Data Table S1). Thus, we only analysed the earliest event between BBCH 11 and 60 for each species, and used the term ‘budburst’ to represent BBCH 11 or BBCH 60.

### Statistical analysis of experimental data

**Fig. 2.**
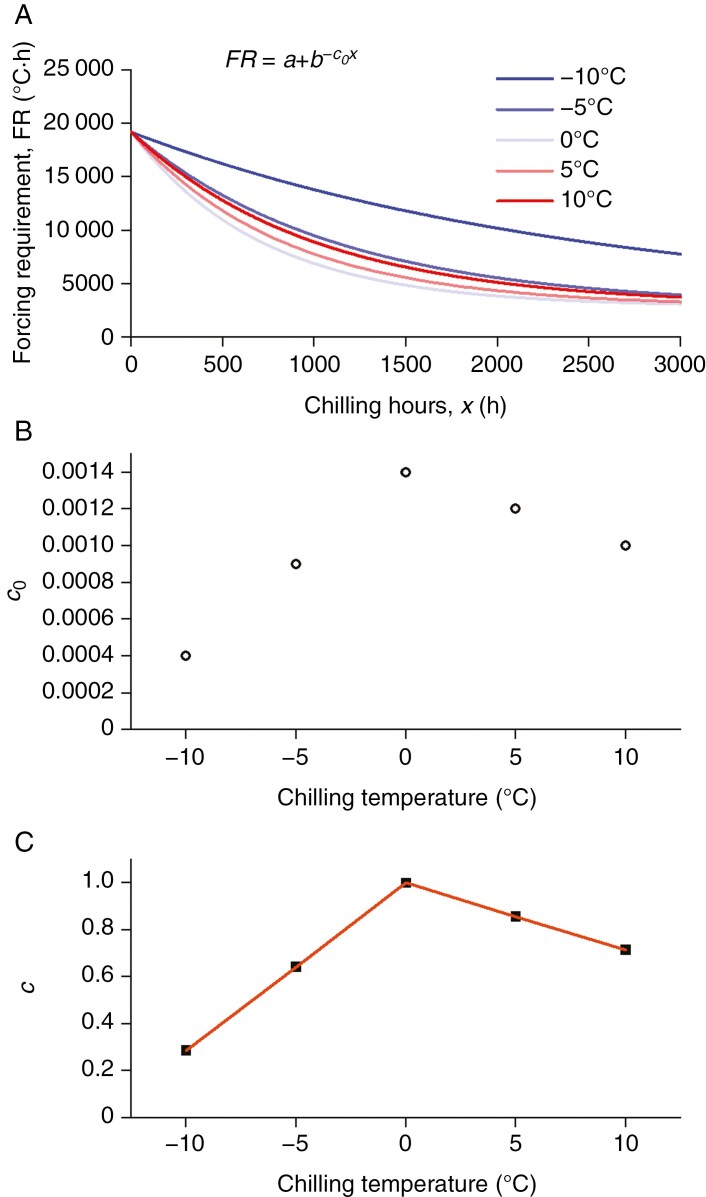
Schematic diagram of the experimental methods to determine chilling function. (A) Fitting the exponential function between the forcing requirement of budburst and chilling hours (Eqn. 1) under different chilling temperatures (*T*). (B) Plotting the parameter *c*_0_ in the exponential function with respect to *T*. (C) Normalizing *c*_0_ to *c* and fitting an empirical function (e.g. a piece-wise linear function).


Based on previous literature (Cannell and Smith, 1983; Nanninga *et al.*, 2017; Baumgarten *et al.*, 2021; Wang *et al.*, 2022*b*), the forcing requirement (FR) of budburst and chilling duration followed a negative exponential curve (Eqn. 1; Fig. 2A).

$$\begin{array}{l} FR=a+be^{-c_{0}(T)\times x} \end{array}$$ (1)

where *a*, *b* and *c*_0_ are model parameters; *a* is the asymptote, *a* + *b* is the intercept and *x* is the chilling duration (h). According to our experimental results, *a* and *b* were very similar among chilling temperatures (*T*) and could be fixed among *T*. *c*_0_ represented the rate of decay and varied among chilling temperatures (*T*). *c*_*0*_(*T*) could reflect the rate of dormancy release under the specific chilling treatment of *T*.

The input data to fit Eqn. (1) included chilling duration *x* and FR of budburst. We first used a growing degree hour (GDH) method to calculate the FR of budburst in the growth chamber. Here, FR (calculated based on the temperature conditions in growth chambers and the number of days before budburst) was used to measure the depth of dormancy.


$$\begin{array}{l} \mathrm{FR}=\sum_{t=t_{s}}^{t_{f}}\max(Tg(t)-Tb,0) \end{array}$$ (2)

where FR represents the total accumulated temperature required for budburst, *t*_s_ refers to the sampling date and *t*_f_ refers to the budburst date (average of five twigs). *T_g_*(*t*) refers to the hourly temperature in the growth chamber. *T*_*b*_ is the threshold temperature for bud development, which is set at 5 °C according to previous studies (Fu *et al.*, 2013; Peaucelle *et al.*, 2019).

Regarding *x*, the chilling hours in refrigerators could not be directly used because our sampling dates were later than the start date of chilling accumulation (CA) in natural conditions. Thus, *x* should be calculated as the sum of chilling hours in refrigerators (CH_R_) and chilling hours received outside (CH_O_) before the sampling date. CH_R_ could be calculated according to the experimental design, while CH_O_ was calculated based on the following assumptions:

(1) The upper threshold of chilling temperatures in the study area is 10 °C. According to previous studies, 1 November was usually used as the date when dormancy begins to release (Cannell and Smith, 1983; Laube *et al.*, 2014; Wang *et al.*, 2020*b*). We thus assumed that dormancy induced by low temperature and short photoperiod had been fully established at this date, and temperature in the following days acts to release dormancy. On 1 November, the daily mean temperature in Beijing was about 10 °C. Therefore, 10 °C is a reasonable upper temperature threshold for dormancy release (see other evidence in the Discussion).

(2) The start date of CA in natural conditions could be determined by the upper threshold of chilling temperature. We assumed that the start date of CA was the first day when the average temperature was <10 °C for the first consecutive 3 d in autumn. Consequently, the start date of CA outside was determined as 3 November 2020 and 4 November 2021. The stable start date of CA between years also indicated that the beginning of CA in natural conditions might also be strongly determined by photoperiod. We used the temperature of three consecutive days rather than 1 d because using a 1-d temperature would lead to a very variable and unreliable start date of CA.

According to our experimental result, the FR of budburst after 30 d of artificial chilling (under five temperature conditions, sampled on 12 December 2020) approximates the FR of budburst after 30 d of natural chilling (Supplementary Data Fig. S2). Thus, exposing buds to natural chilling (temperature fluctuated over time but was always <10 °C) and artificial chilling (a constant <10 °C temperature) with the same duration exerts a similar effect on dormancy release. CH_O_ was then calculated as the number of hours with temperature ≤10 °C from the start date to the sampling date (146 and 367 h in 2020 and 2021, respectively). Finally, *x* for each chilling treatment was calculated as the sum of CH_R_ and CH_O_.

Based on the above processes, the input data to fit Eqn. (1), including FR and x, were calculated for each chilling treatment (Supplementary Data Dataset S1). However, we could not fit Eqn. (1) directly, because it would lead to different a and b among chilling treatments, against our experimental results (different chilling temperatures share the same a and b). Therefore, before fitting Eqn. (1), we pre-set the values of *a* and *b* as 0.95 × min(FR) and 1.1 × max(FR) − *a*, respectively. max(FR) and min(FR) represent the maximum and minimum FR values under all chilling temperatures, respectively. We used this parameter setting because it could better fit the experimental data than other parameter combinations. Finally, we determined *c*_0_ at each chilling temperature by fitting Eqn. (1) based on the least squares method.

To examine whether differences in *c*_0_ were significant between chilling temperatures (*T*), we first took the logarithm of Eqn. (1):


$$\begin{array}{l} ln(FR-a)=-c_{0}(T)\times x+ln(b) \end{array}$$ (3)

According to Eqn. (3), ln(FR − *a*) is the linear function of *x* (chilling duration) and its regression coefficient (*c*_0_) is affected by *T* (chilling temperature). Thus, we constructed a linear fixed-effects model (LFM) with chilling treatments (*T*) and the *x* and *T* interaction (*x* × *T*) as fixed effects:


$$\begin{array}{l} FR_{\ln}∼b_{1}x+b_{2}\left(x\times T\right)+b_{0} \end{array}$$ (4)

where FR_ln_ represents ln(FR − *a*). *b*_0_ is the intercept [representing ln(*b*)], *b*_1_ is the regression coefficient of *x*, representing *c*_0_ under reference chilling temperature (−10 °C), *b*_2_ is the regression coefficient of *x* × *T*, representing *c*_0_ under different *T*. The significance of *b*_2_ is examined by using a *t*-test in the LFM. A significant *b*_2_ (*P* < 0.05) suggests that *c*_0_ under this temperature is significantly different from that of the reference chilling temperature.

### Framework for determining chilling function and associated phenological model

We introduced a new framework for determining chilling functions. Although *c*_0_(*T*) could indicate the rate of dormancy release (Fig. 2B), the maximum rate of dormancy release is usually normalized to 1 so as to construct a chilling function with an arbitrary unit. Thus, the chilling function [*c*(*T*)] is expressed as:


$$\begin{array}{l} c(T)=k\times c_{0}(T) \end{array}$$ (5)

where *k* is a parameter to ensure the maximum value of *c*(*T*) = 1. An empirical function (e.g. piece-wise linear function in Fig. 2C) could be fitted to obtain a continuous *c*(*T*).

In phenological models, CA could be calculated as the accumulated chilling units:


$$\begin{array}{l} CA=c(T)\times x \end{array}$$ (6)

where CA is the chilling accumulation at *T* after *x* hours.

According to Eqns (1), (5) and (6), FR and CA also followed a negative exponential relationship (Eqn. 7).


$$\begin{array}{l} FR=a+be^{-CA/k} \end{array}$$ (7)

Equation (7) is a process-based model with parameters *a*, *b* and *k*. We applied this equation to validate whether the phenological model incorporating chilling functions developed in this study could predict the observed budburst date in experiments and fields. The model input is the hourly temperature data, and the model output is the budburst date or number of days to budburst. This model first used hourly temperature data to calculate CA at a daily time step, and then calculated FR of budburst using Eqn. (7). The day when the actual accumulated GDH exceeds FR was determined as the budburst date.

### Fitting chilling functions based on experimental data

Using the framework above, we fitted a chilling function with a triangular model because *c*_0_(*T*) showed the maximum value at a certain temperature (*T*_op_). We first calculated parameter *k* in Eqn. (5):


$$\begin{array}{l} k=\frac{1}{\max(c_{0}(T))} \end{array}$$ (8)

where $\max(c_{0}(T))$ refers to the maximum value of *c*_0_ among all chilling temperatures.

Based on *k* and Eqn. (5), we calculated *c*(*T*) and fitted a piece-wise linear function (Eqn. 9) using the least squares method:


$$\begin{array}{l} c(T)=\left\{ \begin{array}{llll} & m_{1}\times T+n_{1}\;T\leq Top\; & m_{2}\times T+n_{2}\;Top<T\leq 10\;\; & 0\;T>10\;\; \end{array}\right. \end{array}$$ (9)

where *m*_1_, *n*_1_, *m*_2_ and *n*_2_ are parameters to be fitted and should satisfy Eqn. (10):


$$\begin{array}{l} m_{1}\times T_{op}+n_{1}\;=m_{2}\times T_{op}+n_{2}=1 \end{array}$$ (10)

As mentioned in the Introduction, many previous chilling functions assumed that temperature below an upper threshold has the same effects on dormancy release. This function could be termed the reference function. We used an upper temperature threshold of 10 °C based on the above first assumption and directly constructed a reference chilling function (Eqn. 11):


$$\begin{array}{l} c(T)=\left\{ \begin{array}{l} 1\;T\leq 10\;0\;T>10\; \end{array}\right. \end{array}$$ (11)

When incorporating the reference function in the phenological model, *k* in Eqn. (7) is calculated as the reciprocal of mean *c*_0_(*T*):


$$\begin{array}{l} k=\frac{1}{\bar{c_{0}(T)}} \end{array}$$ (12)

where $\bar{c_{0}(T)}$ is the *c*_0_ averaged from all chilling temperatures.

### Validating chilling function based on the data used to fit the model

We used the observed number of days to budburst as validation data, which encompasses chilling and forcing affecting the model output. A chilling function is more accurate if the phenological model incorporating it could predict the observed budburst date with lower error. Based on this principle, we first validated the chilling functions based on the experimental data used to fit it (training data in Table 2). We used two types of chilling functions (Eqns 9 and 11) to calculate CA, and then used CA, *a*, *b*, *k* and Eqn. (7) to calculate the FR of budburst. The number of days to budburst could be simulated as the result of dividing FR by the thermal time accumulated in the growth chamber per day (380 °C·h). We evaluated the phenological model by calculating *R*^2^, root mean square error (RMSE) and Akaike information criterion with a correction for small sample sizes (AICc) between the simulated and observed number of days to budburst.

### Cross-validation using the external experimental data

To verify whether the chilling function derived from artificial chilling experiments could be used for natural chilling experiments, we used the experimental data under natural chilling (testing data in Table 2) to perform a cross-validation. In natural conditions, temperatures vary over time, and thus Eqn. (6) could not be directly used to calculate CA. Instead, the following equation was used:


$$\begin{array}{l} \mathrm{CA}=\sum_{t=t_{start}}^{t_{end}}c(T_{o}(t)) \end{array}$$ (13)

where *t*_start_ is the first hour of the start date of CA, while *t*_end_ is the last hour of the end date of CA (here *t*_end_ = sampling date). *T*_o_(*t*) is the outside temperature in hour *t*.

In natural conditions, the twigs sampled in early spring (e.g. 14 February 2022) may have accumulated a certain amount of GDHs before moving to the forcing condition. Following previous studies, we assume that the forcing temperature after 1 January may contribute to the accumulated thermal time (Murray *et al.*, 1989; Wang *et al.*, 2020*b*; Lin *et al.*, 2022). Thus, we calculated the accumulated thermal time outside (ΔFR) on each sampling date:


$$\begin{array}{l} \Delta FR=\left\{ \begin{array}{lll} & \sum_{t=t_{1}}^{t_{s}}\max(T_{o}(t)-T_{b},0)ift_{s}\geq t_{1}\; & 0ift_{s}<t_{1}\; \end{array}\right. \end{array}$$ (14)

where *t*_1_ represents 1 January. *T_o_*(*t*) refers to the hourly temperature outside. Other variables have the same meanings as Eqn. (2).

Finally, we used two types of chilling functions (Eqns 6 and 9) to calculate CA based on Eqn. (13), and then used CA, *a*, *b*, *k* and Eqn. (7) to calculate the FR of budburst. The number of days to budburst could be simulated as the result of dividing FR − ΔFR by the thermal time accumulated in the growth chamber per day (380 °C·h). Similarly, *R*^2^, RMSE and AICc were used to compare the simulated and observed number of days to budburst.

### Validation using independent experimental data

We further used the experimental data obtained from Lin *et al.* (2022) to verify the chilling functions. Lin *et al.* (2022) collected twigs of 14 species (including the nine species in this study) 39 times from 1 November 2018 to 26 March 2019 and then moved them to growth chambers to monitor the budburst dates. Similar to the cross-validation, we used the chilling function-based phenological model to simulate the number of days to budburst, and then compared it with the data observed in the experiment.

### Validation using independent observational data

The above validations were all based on experimental data, but whether the chilling function can be used to simulate observational data needed to be verified. We obtained the observational data from the Chinese Phenology Observation Network. The data (spanning 1963–2018) were observed at the Summer Palace, about 16 km from the sampling site of this study. Only seven species (except for *Viburnum dilatatum* and *Cotoneaster horizontalis*) investigated in this study were observed there.

We used two types of chilling functions (Eqns 6 and 9) to calculate CA based on Eqn. (13), and then used CA, *a*, *b*, *k* and Eqn. (7) to calculate the FR of budburst. The budburst date (expressed as day of the year) could be determined as the day when the accumulated thermal time since 1 January first exceeds FR in a given year (examined on a daily basis). Similarly, *R*^2^, RMSE and AICc were used to compare the simulated and observed budburst dates.

## RESULTS

### Effectiveness of freezing temperatures on dormancy release

**Fig. 3.**
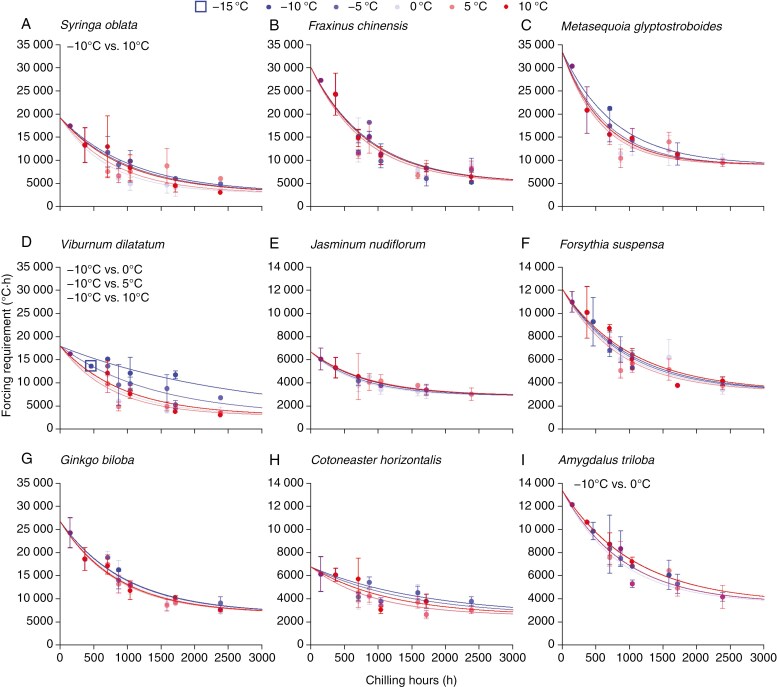
Relationship between forcing requirements of budburst and chilling hours under different chilling temperatures. An exponential curve (Eqn. 1) was fitted for each chilling temperature. Error bar: standard deviation of five replicates.

A wide range of temperatures were effective in releasing dormancy. From short exposure (<200 h) to long exposure (>2000 h) to temperatures at −10 to 10 °C, the FR of budburst and the number of days to budburst decreased rapidly towards the minimum forcing (Fig. 3; Supplementary Data Fig. S3). Freezing temperatures down to −10 °C exerted similar influences on dormancy release as temperature ≥0 °C for most species (Fig. 3), suggesting that freezing temperature could release dormancy as with temperature slightly above zero. For only one species (*V. dilatatum*), freezing temperatures seemed less effective than ≥0 °C temperature (Fig. 3D).

Most species could not budburst in forcing conditions after exposure to a chilling treatment of −15 °C except for *V. dilatatum*. The proportion of budburst (averaged from all chilling durations) and survived twigs were smaller when chilled at −5 and −10 °C, but relatively larger when chilled at 0, 5 and 10 °C (Supplementary Data Fig. S4a, Table S2). Furthermore, the proportion of budburst and survived twigs generally decreased with chilling duration (Supplementary Data Fig. S4b, Table S2).

### Rate of dormancy release in response to different chilling temperatures

For each species, we fitted the exponential curves (Eqn. 1) between the FR of budburst and chilling hours at each chilling temperature (Fig. 3). The parameters (*a*, *b*, *c*_0_) and RMSE of the exponential curves are shown in Supplementary Data Table S3. The exponential curves were able to closely simulate the FR under varying chilling duration and temperatures with a mean RMSE of 1038 °C·h (corresponding to 2.73 d in growth chambers) across all species.

The parameter *c*_0_ of exponential curves could represent the rate of dormancy release under different chilling temperatures. *c*_0_ varied only slightly among different chilling temperatures (Fig. 3). For all species, *c*_0_ was relatively smaller at −10 and −5 °C, then peaked at 0 or 5 °C, and became smaller again at 10 °C. Thus, the most effective chilling temperature probably ranged between 0 and 5 °C. However, the results of LFM indicated that *c*_0_ under the chilling temperature of −10 °C did not differ significantly (*P* > 0.05) from those under other temperatures in most cases (31 out of 36 cases) (Fig. 3). Conversely, chilling duration may have a greater impact on dormancy release than absolute chilling temperatures, because the FR of budburst decreased exponentially with the chilling hours, regardless of the chilling temperatures (Fig. 3). An exception was *V. dilatatum,* for which the rate of dormancy release under −10 °C varied significantly (*P* < 0.05) with those under 0, 5 and 10 °C. Therefore, both the absolute chilling temperatures and chilling hours are important for this species.

### Validation of chilling function-associated phenological models

**Fig. 4.**
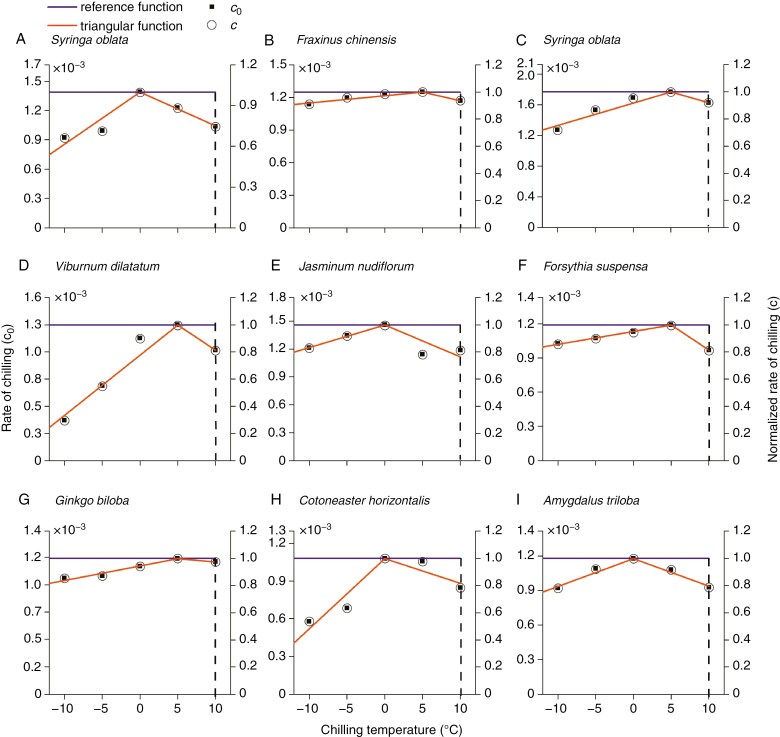
Two types of chilling function for nine temperate species; *c*_0_ (parameter in Eqn. 1) represents the rate of dormancy release (left *y*-axis), and *c*(*T*) represents the normalized rate (chilling unit) under different temperatures (right *y*-axis). The triangular functions were fitted from *c*(*T*), with an upper threshold of 10 °C (vertical dashed line). The reference function was pre-defined based on the assumption that temperature below an upper threshold of 10 °C (vertical dashed line) has the same effects on dormancy release.

We constructed a triangular function by fitting a piece-wise linear function to normalized *c*_0_. We further compared the triangular function with a pre-defined reference function, which assumes different temperatures have the same effects on dormancy release. The curves of chilling functions for each species are shown in Fig. 4. By incorporating two types of chilling functions, we developed corresponding phenological models (Eqn. 7, see parameters in Supplementary DataTable S4).

**Fig. 5.**
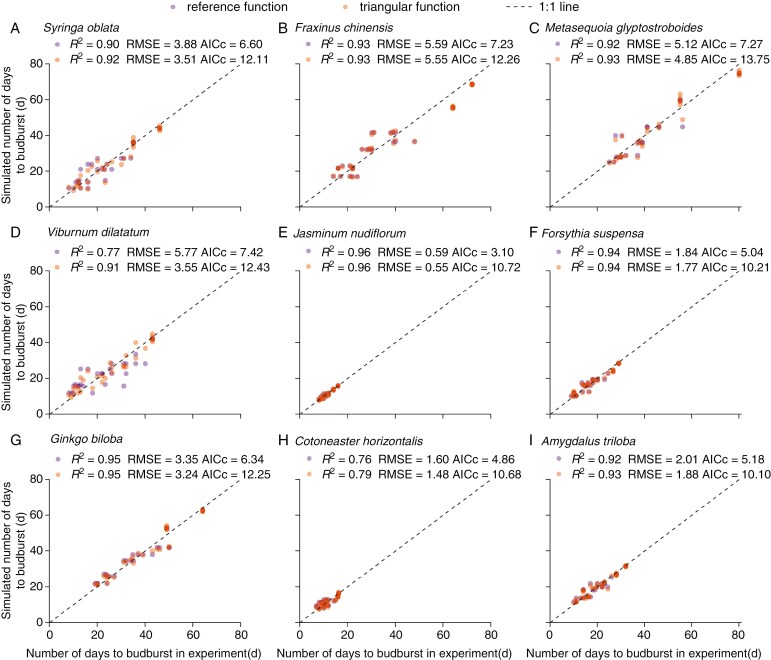
Comparison between simulated and observed number of days to budburst based on the experimental data used to fit chilling functions. The number of days to budburst was simulated by two chilling function-based phenological models.

Regarding internal validation, we found that the scatterplot of the simulated and observed number of days to budburst was distributed around the 1:1 line and correlated significantly with each other (with *R*^2^ ranging from 0.76 to 0.95) for all the species (Fig. 5). The RMSEs of the triangular function-based model (0.55–5.55d) were slightly smaller than the reference function-based model (0.59–5.59 d). However, the AICc of the triangular function-based model (10.10–13.75) was markedly larger than the reference function-based model (3.10–7.42). Thus, when introducing a penalty term for the number of parameters in the model (AICc), the reference function-based model with a smaller number of parameters performed better than the triangular function.

**Fig. 6.**
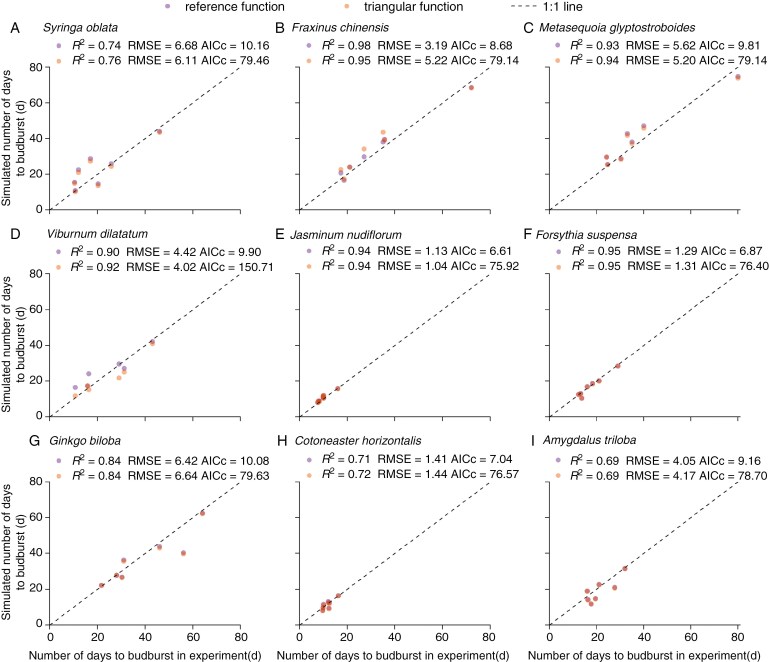
Comparison between simulated and observed number of days to budburst based on the experimental data in 2020 and 2021. The number of days to budburst was simulated by two chilling function-based phenological models.

Furthermore, we used two phenological models to simulate the number of days to budburst for twig exposure to a period of natural chilling in the winter seasons of 2020 and 2021 (cross-validation). Regarding this external dataset, phenological models still could reproduce the number of days to budburst strongly correlating with those observed in the experiments for all species (Fig. 6), but the RMSEs of phenological models increased compared to those in internal validation for several species (such as *Syringa oblata*, *Fraxinus chinensis*, *Ginkgo biloba* and *Amydalus triloba*). The RMSEs of the triangular function-based model were slightly larger than the reference function-based model for five species (*Fr. chinensis*, *Forsythia suspensa*, *G. biloba*, *C. horizontalis* and *A. triloba*). Overall, the outputs of the two models were very close, with most of the points overlapping with each other, but AICc values of the triangular function-based model were larger than those of the reference function-based model (Fig. 6). In addition, we applied the chilling function-based models to independent experimental data collected in the winter season of 2018 (Supplementary DataFig. S5). The results showed that the phenological models could simulate the number of days to budburst observed in an experiment conducted in another year with similar uncertainties, although the error was larger under low chilling conditions (the absolute error reached 20 d) for several species (*V. dilatatum* and *A. triloba*). Likewise, the reference function-based model outperformed the triangular function-based model in terms of AICc for all species, although it showed larger RMSE for three species.

Finally, we compared the budburst dates of seven species simulated by phenological models with the long-term observational data at an adjacent site in Beijing. The simulated budburst dates correlated positively and significantly with the observed dates, with *R*^2^ ranging from 0.44 to 0.82 and RMSE ranging from 2.89 to 16.48 d (Supplementary DataFig. S6). It is worth noting that the scatterplot between the simulated and observed dates deviated from the 1:1 line (RMSE* *> 10 d) for three species (*Metasequoia glyptostroboides*, *Jasminum nudiflorum* and *G. biloba*), possibly because of the phenological differentiation between populations and the difference in microclimate between the observational and experimental site. Based on AICc, the performance of the reference function-based model was better than the triangular function-based model, although the RMSE of the triangular function-based model was 0.04–0.28 d smaller than the reference-based model for seven species (Supplementary Data Fig. S6).

## DISCUSSION

### Effectiveness of freezing temperature on dormancy release

This study demonstrated that freezing temperatures as low as −5 and −10 °C were effective in releasing bud from dormancy for all temperate species investigated (Fig. 3). We also attempted exposing twigs to −15 °C in an initial experiment, but the majority of twigs subjected to this temperature died. The possible reason was that twigs did not receive enough cold acclimation in early November, and −15 °C exceeded their maximum cold hardiness at that time. Also, the duration of exposure to −15 °C reached 13 d in experiments, while in the outdoors Beijing, plants were only exposed to −15 °C for several hours in winter (Supplementary Data Fig. S1). Meanwhile, twigs, separated from the rest of the plant, are more vulnerable to cold than the twigs connected to the branch. In temperate and boreal regions, trees typically survive winter temperatures of −15 °C, and thus it is possible that such temperatures still contribute to dormancy release. Many previous chilling functions assumed that <0 °C did not contribute to dormancy release, for example the functions ADN, IDN, ASN_1_ and ASN_2_ in Table 1. Even for the chilling functions considering the effects of freezing temperature, the lower threshold for effective chilling was often as high as −2 °C (IDF_2_ in Table 1). These chilling functions, derived from an assumed temperature response or inverse modelling technique, were not supported by our experiments.

The effectiveness of freezing temperature on dormancy release was in accordance with many previous experimental studies focusing on species from various regions. For example, placing twigs of two arctic deciduous shrubs (*Salix pulchra* and *Betula nana*) at –5 °C for more than 30 d could advance the budburst date and increase bud break success (Pop *et al.*, 2000). For *Ribes nigrum* in Dundee, UK, –5 °C was more effective in increasing the percentage of budburst than 0, 5 and 10 °C (Jones *et al.*, 2013). Similarly, exposure to −10 °C for 8 weeks released dormancy of *R. nigrum* in South Norway, although continued chilling for another 8 weeks inhibited bud break (Sønsteby and Heide, 2014). In a chilling experiment conducted in Zurich, Switzerland, −2 °C was more effective for satisfying the chilling requirements of three temperate trees than other temperatures ≥0 °C (Baumgarten *et al.*, 2021). Given that so much experimental evidence supports our findings, we suggest that the chilling function should consider the effects of freezing temperatures.

If we chose a chilling function with no lower limit but an upper limit of temperature (e.g. ASF in Table 1), CA calculated from this chilling function would decrease with winter warming because warming would definitely make the temperature during specific periods exceed the upper limit. However, if the chilling function assumes that freezing temperature does not contribute to accumulated chilling and has a lower limit of around 0 °C, CA may increase with warming, as the ineffective below-zero temperature may increase to an effective above-zero temperature (Wang *et al.*, 2020*a*). When analysing the long-term budburst data of *Picea sitchensis* in the UK, using the function EDF_1_ in Table 1 (assuming an optimum chilling temperature of 3.4 °C decreasing to zero effect at both −3.4 and +10.4 °C) produced no discernible relationship between CA and FR, but the function ASF (assuming temperature of <5 °C were equally effective) provided the best-fitting curve between CA and FR (Cannell and Smith, 1983). The ‘<7.2 °C’ chilling function could best explain the interannual changes in the flowering duration of *Rubus idaeu* than the ‘0–7.2 °C’ function (ASN_1_ in Table 1) (Sunley *et al.*, 2006). Furthermore, the chilling functions that do not account for freezing temperatures could not correctly reflect the negative relationship between CA and FR of budburst for 30 perennial species (Wang *et al.*, 2020*a*). These studies based on observational data provided indirect evidence of the effectiveness of freezing temperature in dormancy release.

### Upper temperature threshold for effective chilling

This study assumed that the upper temperature threshold for effective chilling is 10 °C and did not test the effectiveness of >10 °C on dormancy release in the experiment. The upper temperature threshold adopted in this study was a commonly used threshold when experimentally studying the effects of chilling for temperate and boreal species. For example, 10 °C could release the dormancy for apples in Somerset West, South Africa (Cook *et al.*, 2005), *Betula pendula* and *B. pubescens* in Northern Europe (Myking and Heide, 1995), *Ribes* cultivars in the UK and Norway (Jones *et al.*, 2013; Sønsteby and Heide, 2014), and six European trees in Switzerland (Baumgarten *et al.*, 2021).

In addition, the effectiveness of >10 °C on dormancy release of temperate and boreal species has been rarely tested using an explicit experimental design. The effect of above 10 °C (and possibly above 5 °C) can be confused with forcing, which can accumulate simultaneously with chilling, complicating the determination of the upper CA threshold. The only example of >10 °C temperature was from an experiment conducted in Ås, Norway (Heide and Prestrud, 2005). Apple (*Malus pumila*) was unable to resume normal growth for cultivar ‘M9’ or resume growth slowly for cultivar ‘MM106’ after 14 weeks of exposure to 12 °C (Heide and Prestrud, 2005). This finding suggested that a temperature above 10 °C exerts little effect on dormancy release. However, it should be pointed out that the upper temperature threshold of 10 °C used in this study may not be suitable for the subtropical regions (Zhang *et al.*, 2021).

### Similar effects of dormancy release over a wide range of temperatures

The rate of dormancy release (indicated by *c*_0_) was not significant between −10 °C and other temperatures in 31 out of 36 cases (Fig. 3), suggesting that the effects of dormancy release were similar over a wide range of temperatures. Similar results were found in previous studies. For example, no significant differences in chilling efficiency were observed between temperatures of 0, 5 and 10 °C for *B. pendula* and *B. pubescens* (Myking and Heide, 1995) and between temperatures of 1, 4, 7 and 10 °C for apple trees (Cook *et al.*, 2005). A range of temperatures from −2 to 10 °C showed similar effects on the dormancy release of six European trees (Baumgarten *et al.*, 2021). For four subtropical trees in China, no significant difference or pattern in chilling effects was found among temperatures of 5, 10 and 15 °C (Zhang *et al.*, 2021). The indirect evidence was the reference function-based model (assuming an equal effect over a range of temperatures) could reproduce the interannual changes of budburst dates in the field with a lower RMSE than other models (Supplementary DataFig. S6).

Although the rate of dormancy release was similar over a wide range of temperatures, we found that one species (*V. dilatatum*) exhibited significantly different *c*_0_ among chilling temperatures. Thus, the varying effects of chilling temperature among temperatures exist for certain species. For instance, Sarvas (1974) found that *B. pubescens* showed the maximum chilling effect at 3.5 °C and zero effect at −3.4 and 10.4 °C. For *B. pendula* in Northern Europe, the days needed to budburst were fewer after >30 d of chilling at 6 °C than those at 0 and 3 °C (Skuterud and Dietrichson, 1994). Chilling at −5 °C for 14 weeks or more was optimal for breaking dormancy than other temperatures (−10, 0, 5 and 10 °C) for most cultivars of *R. nigrum* (Sønsteby and Heide, 2014).

### Framework for developing chilling function and phenological model

In chilling–forcing experiments, dormancy status could be reflected by two indicators: the proportion of budburst or the number of days to first budburst (Hänninen, 2016). With longer chilling duration, the proportion of budburst observed in forcing conditions increases, while the number of days to budburst usually decreases. In previous phenological models, the determination of chilling functions could rely on one of these two indicators.

Based on the proportion of budburst, the rate of dormancy release could be calculated as the reciprocal of the development time (ΔT) required for dormancy completion. The criterion of dormancy completion used in Sarvas (1974) was the proportion of budburst reached 50 % after 21 d in forcing conditions. Another example was Jones *et al.* (2013), which used the rate of increase in the proportion of budburst with chilling duration to determine the chilling function. In this study, although we recorded the proportion of budburst in the experiment, we did not use it to determine the chilling function because the sample size (usually about 3–6 buds per twig) was not enough to generate a smooth budburst curve over time.

Another method to determine the chilling function was based on the number of days to budburst under different chilling temperatures. For example, Zhang *et al.* (2022) defined ΔT as the days required for the number of days to budburst levelling off (first derivative of the exponential curve = −0.3), and then the chilling function was determined as the reciprocal of ΔT.

In this study, we adopted a novel method to determine the chilling function. First, we replaced the number of days to budburst in the previous method with the FR of budburst to determine the rate of dormancy release. Forcing conditions adopted in experiments would lead to a very different number of days to budburst. For example, for dormant twigs of cherry plum (*Prunus cerasifera* ‘Atropurpurea’), the number of days to budburst at a forcing temperature of 15 °C (19 d) was almost double that at 25 °C (10 d), but the FR (with a base temperature of 5 °C) at these two temperatures were similar (190 vs. 200 degree-days) (Wang *et al.*, 2021). Thus, the curve between the number of days to budburst and chilling duration would be affected by forcing conditions, and the rate of chilling derived from one experiment could not be compared with another one, as different experiments often use distinct forcing conditions. Second, we directly used the parameter *c*_0_ of the exponential function to represent the rate of dormancy release rather than ΔT because this method could further derive a chilling function-based phenological model (Eqn. 7).

The validation results confirmed that our phenological models not only could reproduce experimental budburst date using the model (Fig. 5), but also could predict the number of days to budburst in external experiments (Fig. 6; Supplementary Data Fig. S4) with a similar RMSE (0.55–8.53 d) in other modelling studies (Fu *et al.*, 2012; Gauzere *et al.*, 2017). Interestingly, the triangular function-based model considering different effects of temperatures predicted almost the same budburst date as the reference function-based model when simulating phenology in natural conditions (Supplementary Data Fig. S6). This further confirmed the effectiveness of freezing temperature on dormancy release and similar effects over a wide range of temperatures. Thus, when developing spring phenological models for other temperate and boreal species lacking experimental data, we recommend directly adopting the reference function developed in this study rather than using the inverse modelling technique. Although different species can share the same chilling function to calculate CA, species-specific experiments are still required to determine the parameter *k* in Eqn. (7). However, for species such as *V. dilatatum*, using the reference function may introduce great uncertainties, as their rates of dormancy release vary significantly with chilling temperatures.

We noted that species with earlier spring phenology (lower FR), such as *J. nudiflorum*, *Fo. suspensa*, *C. horizontalis* and *A. triloba*, had better prediction accuracy (lower RMSE) than the other five species with relatively higher FR (Figs 5 and 6). The main reason is that for species with lower FR, the simulation error of budburst date was smaller than that for species with higher FR when the model has the same relative error of FR. Furthermore, the spring phenology of these early-season species (usually shrubs) was relatively easy to predict because their budburst dates closely followed the temperature changes in order to obtain resources before the canopy was closed (Panchen *et al.*, 2014). However, the late-season species (usually trees) take a long time to budburst. Thus, the prediction error of the model based on chilling and forcing temperature may increase due to the influence of other environmental factors (e.g. water and light conditions) during the process of ontogenetic development.

Considering that previous studies often used five or fewer replicates to conduct the chilling experiment (Jones *et al.*, 2013; Baumgarten *et al.*, 2021; Lin *et al.*, 2022), we directly adopted five replicates. However, less than half of the twigs survived to budburst under treatment of −10 °C with any chilling duration (Supplementary Data Table S2), reducing the robustness of our results. Thus, the number of replicates may not be enough and needs to be increased in future experiments, especially for experiments focusing on chilling temperature down to −10 °C. Previous phenological models assumed that daylength is an additive or multiplicative factor affecting the rate of chilling (Campbell and Sugano, 1975; Kramer, 1994). However, a recent experiment indicated that the photoperiod (0, 8 or 16 h) received during the chilling treatment (at 10 °C) did not affect the budburst of *B. pubescens* (Caffarra *et al.*, 2011). Thus, in this study, we chilled the twigs in full darkness. Further studies are necessary to assess whether the insignificant effects of photoperiod in releasing bud dormancy are true for most temperate species (Heide, 1993; Caffarra *et al.*, 2011). Furthermore, we noted that the proportion of budburst and survived twigs generally decreased with the chilling duration (Supplementary Data Fig. S4a, Table S2), possibly because prolonged storage caused dehydration of twigs. Certain measures need to be taken to prevent dehydration during experiments (e.g. increasing air humidity).

## CONCLUSIONS

In summary, we have demonstrated that freezing temperatures of down to −10 °C are effective in dormancy release for the temperate species examined, contrary to the well-known assumption of <0 °C temperature generally not contributing to accumulated chilling in previous chilling models. Furthermore, the rate of dormancy release did not differ significantly among chilling temperatures in most cases, although it exhibited a maximum value at 0 or 5 °C, and decreased slightly at −10, −5 and 10 °C. We fitted a triangular function based on the experimental data and compared it with a pre-defined reference function. The phenological models incorporating either chilling function could simulate the number of days to budburst in experiments and budburst dates in observational data with a mean RMSE of 7.07 d. Future studies could apply a similar experiment and framework proposed in this study to develop the species-specific chilling function and associated spring phenological models.

## SUPPLEMENTARY DATA

Supplementary data are available at *Annals of Botany* online and consist of the following.

Dataset S1: the experimental data derived in this study. Table S1. Summary of woody species investigated in this study. Table S2. The proportion of twigs that could reach the budburst stage (BBCH 9) after exposure to different chilling treatments. Table S3. Parameters of the exponential function between chilling hours and forcing requirement of budburst. Table S4. Parameters of chilling function-associated phenological models. Figure S1. Daily mean, maximum and minimum temperature over two winter seasons in Beijing. Figure S2. Comparisons of forcing requirement of budburst date among the same duration (30 d) of natural and artificial chilling at different temperatures across all species. Figure S3. Relationship between number of days to budburst and chilling hours under different chilling temperatures. Figure S4. Proportion of budburst under different chilling temperatures and hours. Figure S5. Comparison between the simulated and observed number of days to budburst based on an independent experimental dataset. Figure S6. Comparison between simulated and observed budburst date based on long-term observations from an adjacent site.

mcae112_suppl_Supplementary_Material

mcae112_suppl_Supplementary_Data
