# Supplementary material for: Effectiveness of freezing temperatures on dormancy release of temperate woody species
Source: Ann Bot. 2024 Jul 25;134(4):615–30. doi: 10.1093/aob/mcae112 (PMC11523621; doi:10.1093/aob/mcae112)
Supplement: mcae112_suppl_Supplementary_Material [file mcae112_suppl_supplementary_material.docx]

**Supplementary Materials**

**Table S1** Summary of woody species investigated in this study

| No. | Species | Common Name | Life form | Budburst |
| --- | --- | --- | --- | --- |
| 1 | *Syringa oblata* | Early blooming lilac | DBS | FLD |
| 2 | *Fraxinus chinensis* | Chinese ash | DBT | FLD |
| 3 | *Metasequoia glyptostroboides* | Dawn redwood | DCT | FLD |
| 4 | *Viburnum dilatatum* | Linden arrowwood | DBS | FLD |
| 5 | *Jasminum nudiflorum* | Winter jasmine | DBS | FFD |
| 6 | *Forsythia suspensa* | Golden-bell | DBS | FFD |
| 7 | *Ginkgo biloba* | Ginkgo | DBT | FLD |
| 8 | *Cotoneaster horizontalis* | Rockspray | DBS | FLD |
| 9 | *Amygdalus triloba* | Flowering plum | DBS | FFD |

DBS: deciduous broadleaved shrub; DBT: deciduous broadleaved tree; DCT, deciduous coniferous tree. FLD, first leaf date (BBCH 11); FFD: first flowering date (BBCH 60)

**Table** **S2** The proportion of twigs that could reach the budburst stage (BBCH 9) after exposing to different chilling treatments

| Chilling temperatures | Chilling duration | | | | | | |
| --- | --- | --- | --- | --- | --- | --- | --- |
|  | Sampled on 12 Nov. 2020 | | | Sampled on 22 Nov. 2021 | | | |
|  | 13 days | 30 days | 60 days | 14 days | 28 days | 56 days | 84 days |
| −15°C | 0.022 | 0 | 0 | NA | NA | NA | NA |
| −10°C | 0.200 | 0.133 | 0.022 | 0.356 | 0.311 | 0.156 | 0.067 |
| −5°C | NA | 0.511 | 0.289 | 0.733 | 0.733 | 0.667 | 0.400 |
| 0°C | NA | 0.711 | 0.844 | 0.800 | 0.778 | 0.689 | 0.422 |
| 5°C | NA | 0.911 | 0.667 | 0.867 | 0.778 | 0.667 | 0.600 |
| 10°C | NA | NA | NA | 0.867 | 0.644 | 0.489 | 0.333 |

The value in this table was the mean of 9 species investigated. NA: not applicable.

Table S3 Parameters of the exponential function between chilling hours and forcing requirement of budburst

| Species | Temperature  (℃) | *a* | *b* | *c_0_* | *RMSE* (°C·h) | *RMSE_n_*  (d) |
| --- | --- | --- | --- | --- | --- | --- |
| *Syringa oblata* | -10 | 2888 | 16340±1979.63 | 0.000920965±0.0003716 | 846 | 2.23 |
|  | -5 | 2888 | 16340±1979.63 | 0.000990435±0.0003657 | 591 | 1.56 |
|  | 0 | 2888 | 16340±1979.63 | 0.001387134±0.0003626 | 1315 | 3.46 |
|  | 5 | 2888 | 16340±1979.63 | 0.001227056±0.0003626 | 1857 | 4.89 |
|  | 10 | 2888 | 16340±1979.63 | 0.001035773±0.0003679 | 1170 | 3.08 |
| *Fraxinus chinensis* | -10 | 5054 | 25042±3886.78 | 0.001137659±0.0001729 | 1442 | 3.79 |
|  | -5 | 5054 | 25042±3886.78 | 0.001198354±0.0002051 | 2910 | 7.66 |
|  | 0 | 5054 | 25042±3886.78 | 0.001232238±0.0001980 | 2006 | 5.28 |
|  | 5 | 5054 | 25042±3886.78 | 0.001251504±0.0001954 | 2229 | 5.87 |
|  | 10 | 5054 | 25042±3886.78 | 0.001171506±0.0002088 | 1523 | 4.01 |
| *Metasequoia glyptostroboides* | -10 | 9025 | 24415±2744.90 | 0.001273135±0.0005392 | 2460 | 6.47 |
|  | -5 | 9025 | 24415±2744.90 | 0.00153402±0.0005292 | 1278 | 3.36 |
|  | 0 | 9025 | 24415±2744.90 | 0.001689828±0.0005166 | 1645 | 4.33 |
|  | 5 | 9025 | 24415±2744.90 | 0.001769032±0.0005166 | 2191 | 5.77 |
|  | 10 | 9025 | 24415±2744.90 | 0.001628788±0.0005292 | 1474 | 3.88 |
| *Viburnum dilatatum* | -10 | 2888 | 15086±2034.42 | 0.000385717±0.0001830 | 1151 | 3.03 |
|  | -5 | 2888 | 15086±2034.42 | 0.000713792±0.0001802 | 1277 | 3.36 |
|  | 0 | 2888 | 15086±2034.42 | 0.001168761±0.0001802 | 1380 | 3.63 |
|  | 5 | 2888 | 15086±2034.42 | 0.001295214±0.0001802 | 1331 | 3.50 |
|  | 10 | 2888 | 15086±2034.42 | 0.001054795±0.0001881 | 1291 | 3.40 |
| *Jasminum nudiflorum* | -10 | 2888 | 3800±358.61 | 0.001211386±0.0006934 | 6 | 0.02 |
|  | -5 | 2888 | 3800±358.61 | 0.001342589±0.0006731 | 123 | 0.32 |
|  | 0 | 2888 | 3800±358.61 | 0.00145622±0.0006732 | 303 | 0.80 |
|  | 5 | 2888 | 3800±358.61 | 0.001141099±0.0006732 | 143 | 0.38 |
|  | 10 | 2888 | 3800±358.61 | 0.001186625±0.0007077 | 20 | 0.05 |
| *Forsythia suspensa* | -10 | 3249 | 8873±1258.50 | 0.001021177±0.0003176 | 608 | 1.60 |
|  | -5 | 3249 | 8873±1258.50 | 0.001070074±0.0003070 | 542 | 1.43 |
|  | 0 | 3249 | 8873±1258.50 | 0.001124121±0.0003032 | 755 | 1.99 |
|  | 5 | 3249 | 8873±1258.50 | 0.001185573±0.0003032 | 715 | 1.88 |
|  | 10 | 3249 | 8873±1258.50 | 0.00096622±0.0003098 | 694 | 1.83 |
| *Ginkgo biloba* | -10 | 6859 | 19893±1649.09 | 0.001018555±0.0002616 | 1338 | 3.52 |
|  | -5 | 6859 | 19893±1649.09 | 0.001037554±0.0002578 | 1393 | 3.67 |
|  | 0 | 6859 | 19893±1649.09 | 0.001122146±0.0002533 | 1397 | 3.68 |
|  | 5 | 6859 | 19893±1649.09 | 0.001190966±0.0002533 | 1030 | 2.71 |
|  | 10 | 6859 | 19893±1649.09 | 0.001161412±0.0002563 | 986 | 2.59 |
| *Cotoneaster horizontalis* | -10 | 2527 | 4244.6±773.41 | 0.000580213±0.0015285 | 210 | 0.55 |
|  | -5 | 2527 | 4244.6±773.41 | 0.00068658±0.0014920 | 569 | 1.50 |
|  | 0 | 2527 | 4244.6±773.41 | 0.001080494±0.0014920 | 610 | 1.61 |
|  | 5 | 2527 | 4244.6±773.41 | 0.001056415±0.0014920 | 393 | 1.03 |
|  | 10 | 2527 | 4244.6±773.41 | 0.000847181±0.0014944 | 712 | 1.87 |
| *Amygdalus triloba* | -10 | 3610 | 9766±1002.27 | 0.000922487±0.0001553 | 256 | 0.67 |
|  | -5 | 3610 | 9766±1002.27 | 0.001090294±0.0001514 | 613 | 1.61 |
|  | 0 | 3610 | 9766±1002.27 | 0.001179242±0.0001514 | 1087 | 2.86 |
|  | 5 | 3610 | 9766±1002.27 | 0.001082324±0.0001514 | 779 | 2.05 |
|  | 10 | 3610 | 9766±1002.27 | 0.000929253±0.0002538 | 74 | 0.19 |

For each species and a chilling temperature, parameters (*a*, *b*, *c_0_*) of an exponential function (Eqn. 1) were fitted. The values after “±” represent the standard error (SE) estimated by the linear fixed-effects model (Eqn. 4). *RMSE* is the root mean square error of the exponential function for simulating the forcing requirements of budburst in the experiments. *RMSE_n_*: root mean square error of the exponential function for simulating the number of days to budburst in growth chambers.

Table S4 Parameters of chilling function-associated phenological models

| NO. | Species | Reference function (Eqn. 6) | Triangle function (Eqn. 9) | | | | | |
| --- | --- | --- | --- | --- | --- | --- | --- | --- |
|  |  | *k* | *T_op_* | *m_1_* | *n_1_* | *m_2_* | *n_2_* | *k* |
| 1 | *Syringa oblata* | 899.060 | 0 | 0.0383 | 1 | -0.0249 | 1 | 720.911 |
| 2 | *Fraxinus chinensis* | 834.549 | 5 | 0.0053 | 0.973 | -0.0128 | 1.064 | 799.039 |
| 3 | *Metasequoia glyptostroboides* | 633.328 | 5 | 0.0164 | 0.918 | -0.0159 | 1.079 | 565.281 |
| 4 | *Viburnum dilatatum* | 1082.655 | 5 | 0.0443 | 0.778 | -0.0371 | 1.186 | 772.073 |
| 5 | *Jasminum nudiflorum* | 788.903 | 0 | 0.0166 | 1 | -0.0235 | 1 | 686.710 |
| 6 | *Forsythia suspensa* | 931.591 | 5 | 0.0095 | 0.953 | -0.0370 | 1.185 | 843.474 |
| 7 | *Ginkgo biloba* | 904.056 | 5 | 0.0107 | 0.946 | -0.0050 | 1.025 | 839.655 |
| 8 | *Cotoneaster horizontalis* | 1176.226 | 0 | 0.0516 | 1 | -0.0182 | 1 | 925.503 |
| 9 | *Amygdalus triloba* | 960.873 | 0 | 0.0204 | 1 | -0.0202 | 1 | 848.002 |

*k* is the parameter in the phenological model (Eqn. 7) for reference or triangular function. *T_op_*, *m_1_*, *n_1_*, *m_2_*, *n_2_* are parameters in Eqn. (9).





**Figure S1** Daily mean, maximum and minimum temperature over two winter seasons in Beijing. The red points mark the sampling dates in the field.





**Figure S2** Comparisons of the forcing requirement for budburst among the same duration (30 d) of natural and artificial chilling at different temperatures across all species. Each point represents the mean of 5 replicates for each species in each chilling treatment. Error bar: standard deviation.





Figure S3 Relationship between number of days to budburst and chilling hours under different chilling temperatures. Error bar: standard deviation of 5 replicates.





**Figure S4** Proportion of budburst under different chilling temperatures and hours. (a) comparison among different chilling temperatures. (b) comparison among different chilling hours. The chilling hours are the sum of duration under natural chilling before 22 November 2021 (367 h) and under artificial chilling. Bottoms and tops of boxes: 25th and 75th percentiles; bands within boxes: medians; whiskers: 10th and 90th percentiles; circles: mean value over the entire distribution.





**Figure S5** Comparison between the simulated and observed number of days to budburst based on an independent experimental dataset. The experimental data (winter season of 2018) were derived from Lin *et al.* (2022). The number of days to budburst was simulated by two chilling function-based phenological models.





**Figure S6** Comparison between simulated and observed budburst date based on long-term observations from an adjacent site. The budburst date (in day of the year, DOY) was simulated by two chilling function-based phenological models.
